# Electrochemical behavior of 2205 duplex stainless steel in simulated solution containing high concentration Cl^−^ and saturated CO_2_ at different temperatures

**DOI:** 10.1038/s41598-022-16096-4

**Published:** 2022-07-12

**Authors:** Yanpeng Li, Shidong Zhu, Jun Xian, Xuanpeng Li, Yuqin Zhao, Shusen Wang

**Affiliations:** 1 School of Materials Science and Engineering, Xi’an Shiyou University, Xi’an, 710065 China; 2 State Key Laboratory for Performance and Structure Safety of Petroleum Tubular Goods and Equipment Materials, CNPC Tubular Goods Research Institute, Xi’an, 710077 China; 3 Department of Ground Engineering of PetroChina Tarim Oilfield Company, Korla, 841000 Xinjiang China; 4 Technical Research Center of No. 1 Gas Production Plant, PetroChina Qinghai Oilfield Company, Qinghai, 816000 China

**Keywords:** Corrosion, Electrochemistry

## Abstract

2205 duplex stainless steel (DSS) has good corrosion resistance due to its typical duplex organization, but the increasingly harsh CO_2_-containing oil and gas environment leads to different degrees of corrosion, especially pitting corrosion, which seriously threatens the safety and reliability of oil and gas development. In this paper, the effect of temperature on the corrosion behavior of 2205 DSS in a simulated solution containing 100 g/L Cl^−^ and saturated CO_2_ was investigated with immersion tests and electrochemical tests and combined with characterization techniques such as laser confocal microscopy and X-ray photoelectron spectroscopy. The results show that the average critical pitting temperature of 2205 DSS was 66.9 °C. When the temperature was higher than 66.9 °C, the pitting breakdown potential, passivation interval, and self-corrosion potential decreased, while the dimensional passivation current density increased, and the pitting sensitivity was enhanced. With a further increase in temperature, the capacitive arc radius of 2205 DSS decreased, the film resistance and charge transfer resistance gradually decreased, the carrier density of the donor and acceptor in the product film layer with n + p bipolar characteristics also increased and the inner layer of the film with Cr oxide content decreased, while the outer layer with Fe oxide content increased, the dissolution of the film layer increased, the stability decreased, and the number and pore size of pits increased.

## Introduction

With the rapid development of economic and social progress, the increasing demand for oil and gas resources has forced the development of oil and gas to the gradually harsher conditions and environment of the southwest China and the sea, so the service conditions of downhole tubing are becoming more and more severe^1–3^. In the field of oil and gas exploration, the increasing CO_2_ content^4^ and mineralization and Cl^-^ ion content^5,6^ in the produced fluids causes severe corrosion of ordinary carbon steel tubing^7^, even if the injection of corrosion inhibitors into the tubing column can`t effectively inhibit corrosion, carbon steel can no longer meet the long-term service requirements in harsh CO_2_ corrosive environment^8–10^. Research workers have shifted their targets to duplex stainless steel (DSS) with better corrosion resistance. 2205 DSS, with approximately 50% each of ferrite and austenite content in the steel, has excellent mechanical properties and corrosion resistance, a dense surface passivation film, excellent resistance to uniform corrosion, and lower price compared to that of nickel-based alloys^11,12^. Therefore, 2205 DSS was usually used as pressure vessels serving in harsh corrosive environments, oil well casing in CO_2_ corrosive environments, and water coolers for condensing systems in petroleum and chemical marine fields^13–15^, but 2205 DSS may also suffer from corrosion perforation during its use.

At present, there are more studies related to CO_2_ corrosion and Cl^-^ induced pitting corrosion of 2205 DSS at domestic and foreign^16–18^. Ebrahimi^19^ found that the addition of potassium dichromate salt to NaCl solution inhibited the occurrence of pitting corrosion of 2205 DSS, and the increasing concentration of potassium dichromate salt increased the critical pitting temperature of 2205 DSS. While the pitting potential of 2205 DSS increased due to the addition of a quantitative concentration of NaCl to potassium dichromate, and decreased with the increase of NaCl concentration. Han^20^ showed that when the temperature was between 30 and 120 °C, the structure of the 2205 DSS passivation film consisted of a mixture of inner Cr_2_O_3_, outer FeO and Cr-rich; while when the temperature was increased to 150 °C, the passivation film appeared to dissolve, the inner structure was transformed into Cr_2_O_3_ and Cr(OH)_3_, and the outer layer was transformed into Fe(II, III) oxides and Fe (III) hydroxides. Peguet^21^ found that the onset of steady-state pitting of S2205 stainless steel in NaCl solution did not occur generally below the critical pitting temperature (CPT), but within the transition temperature interval (TTI). Tiadi^22^ concluded that the corrosion resistance of S2205 DSS significantly decreased with increasing NaCl concentration, and the more negative the applied potential, the worse the corrosion resistance of the material.

In this paper, the influence of high mineralization, high Cl^−^ concentration and temperature on the corrosion behavior of 2205 DSS was investigated by using characterization means such as dynamic potential scanning, impedance spectroscopy, constant potential, Mott-Schottky curve test and optical electronic microscope and photoelectron spectroscopy, in order to provide theoretical support for the safe service of 2205 DSS in CO_2_-containing oil and gas environment.

## Test materials and methods

### Material and solution

The test material was selected from solid solution treated 2205 DSS (110 ksi steel grade), the main chemical composition was shown in Table 1.

**Table 1:** 2205 DSS main chemical composition.

Element	Cr	Ni	Mo	Mn	Si	C	P	Fe
Content (wt.%)	22.68	4.91	3.46	0.66	0.35	0.015	0.224	balance

The corrosion medium was a simulated solution, prepared from the ions shown in Table 2.

### Test method

Electrochemical specimen size was 10 mm × 10 mm × 5 mm, cleaned with acetone to remove oil and anhydrous ethanol, and blown dry. The back of the specimen is connected with the appropriate length of copper wire by brazing, and after welding, use a multimeter (VC9801A) to test the conductivity of its welded specimen, and then use epoxy resin to seal the non-working surface. The sealed specimen will be polished by 400^#^, 600^#^, 800^#^, 1200^#^, 2000^#^ water-grinding SiC sandpaper and polished on the polishing machine using 0.25 μm polishing agent on the working surface until the surface roughness *R*_a_ ≤ 1.6 μm, and finally cleaned and put into the thermostat.

A Priston (P4000A) electrochemical workstation with a three-electrode system was used, and the auxiliary electrode was a platinum electrode (Pt) with an area of 1 cm^2^, 2205 DSS was the working electrode (with an area of 1 cm^2^), and the reference electrode was (Ag/AgCl). The simulated solutions used in the test were prepared by (Table 2), and the solution was de-oxygenated by passing high purity N_2_ (99.99%) into the solution for 1 h before the test, followed by passing 30 min CO_2_ into the solution, and the CO_2_ in the solution was always saturated during the test.

**Table 2:** Simulated solution ions.

Ions	K^+^	Na^+^	Ca^2+^	Mg^2+^	SO_4_^2−^	HCO^3^^−^	Cl^−^
Concentration (mg/L)	435	55,498	8310	561	430	189	100,000

#### Critical pitting temperature

First, the specimen was placed in a cell with test solution into a thermostat water bath, the initial facility temperature was 2 °C, and the temperature was controlled to rise 1 °C/min, and the temperature range was controlled from 2 to 80 °C. The test was started at a constant potential of (− 0.6142 Vs. Ag/AgCl), and the test curve was the I-t curve, and according to the critical pitting temperature test standard, it is known that when the I-t curve. The temperature when the current density raise to 100 μA/cm^2^ was called the critical pitting temperature. The average critical pitting temperature was 66.9 °C. The test temperatures for the polarization curve and impedance spectrum were selected as 30 °C, 45 °C, 60 °C, and 75 °C, respectively, and the test was repeated three times under the same specimen conditions in order to reduce the possible deviations.

#### Dynamic potential polarization

First, the metal specimens exposed in solution were polarized at cathodic potential (− 1.3 V) for 5 min before the kinetic potential polarization curve test, to eliminate the oxide film formed on the working surface of the specimens, after which the specimens were tested at open circuit potential for 1 h until the corrosion voltage reached a steady state. The scanning speed of the dynamic potential polarization curve test was set to 0.333 mV/s, and the scanning interval potential was set from − 0.3 to 1.2 Vvs.OCP. To ensure the accuracy of the test, the same test conditions were repeated three times.

#### AC impedance

The impedance spectrum test software was Versa Studio. The test was first carried out with the open circuit potential in a steady state, the AC disturbance voltage amplitude was set to 10 mV, the measurement frequency was set to 10^–2^ ~ 10^5^ Hz, and the impedance spectrum data after the test was fitted with ZSimDeme software.

Current–time curve test processes: Different passivation potentials were selected according to the results of the anodic polarization curve, and the I-t curve was measured at a constant potential, and a double logarithmic curve was fitted to calculate the slope of the fitted curve to analyze the film formation mechanism of the passivated film.

#### Semiconductor properties

After the open-circuit voltage was stabilized, the Mott-Schottky curve test was performed. The test potential scan range was 1.0 ~  − 1.0 V (v_S_. Ag/AgCl), the scan rate was 20 mV/s, the test frequency was set to 1000 Hz, and the excitation signal was 5 mV.

### Morphology characterization

X-ray photoelectron spectroscopy (XPS) (ESCALAB 250Xi, UK) was used to sputter-type test the composition and chemical state of the surface passivation film of 2205 DSS after film preparation, and the measured data were processed by peak splitting and fitting using advantage software, compared with the atomic spectrum database and relevant literature^23^, and calibrated with C1s (284.8 eV). And corrosion morphology and pit depth of the tested specimens was characterized using an ultra-deep field optical digital microscope (Zeiss Smart Zoom5 type, Germany).

## Results and discussion

### Critical pitting temperature (CPT)

**Figure 1:**
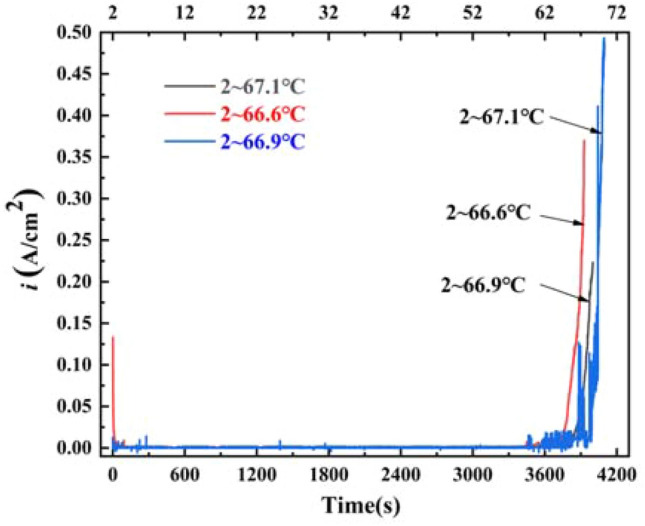
Critical pitting temperature curve of 2205 duplex stainless steel in simulated solution containing 100 g/L Cl^−^ and saturated CO_2_.

The constant potential method was used to test the specimen at the same potential (− 0.6142 Vvs._Ag/AgCl_), and the corrosion current versus time curve was record. According to CPT test standards, the polarization current density increased gradually with the increase in temperature, when the change in polarization current density in the curve was 100 μA/cm^2^, the corresponding temperature was the critical pitting temperature^24,25^. Figure 1 shows the critical pitting temperature of 2205 DSS in simulated solution containing 100 g/L Cl^−^ and saturated CO_2_. It can be seen when the solution temperature was low, the current density was almost unchanged with the extension of the test time. And when the solution temperature increased to a certain value, the current density increased rapidly, indicating that the dissolution rate of the passivation film was increasing with the increase of the solution temperature. When the solution temperature increased from 2 to about 67 °C, the polarization current density of 2205 DSS increased to 100 μA/cm^2^ and the average critical pitting temperature of 2205 DSS was 66.9 °C, which was about 16.6 °C higher than the standard critical pitting temperature of 3.5 wt% NaCl (0.7 V)^26^, which was related to the potential applied at the time of measurement, the lower the applied potential, the higher the measured critical pitting temperature.

### Alternating current impedance (EIS)

**Figure 2:**
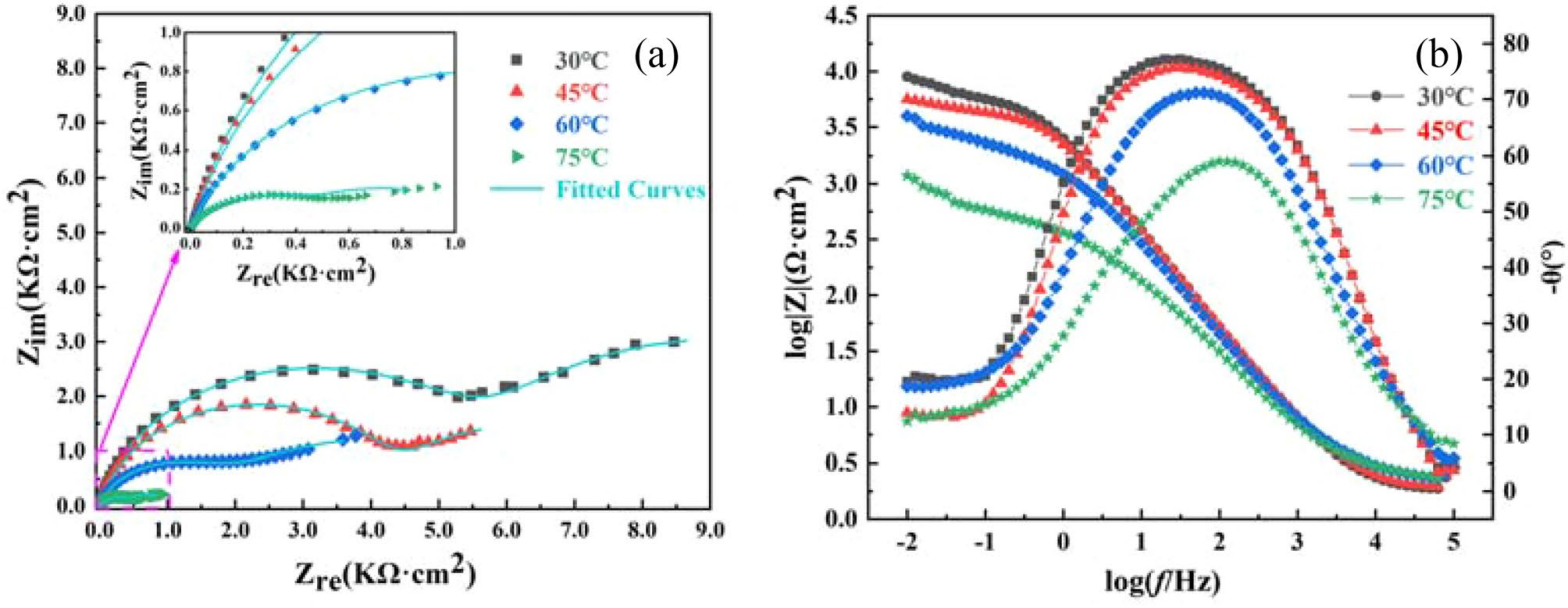
EIS curves of 2205 DSS at different temperatures (**a**) Nyquist plot, (**b**) Bode plot.

Figure 2 shows the AC impedance plots of 2205 DSS in simulated solution containing 100 g/L Cl^−^ and saturated CO_2_ at different temperatures. It can be seen that the Nyquist plots of 2205DSS at different temperatures were composed of high, medium and low frequency capacitive resistance arcs, and the capacitive resistance arcs were non-semi-circular. The radius of the capacitive arc reflected the size of the passivation film resistance and the size of the charge transfer resistance during the electrode reaction. It was generally believed that the larger the radius of the capacitive arc, the better the corrosion resistance of the metal substrate in solution^27^. When the solution temperature was 30 °C, the radius of the capacitive arc in the Nyquist plot and the impedance modulus |Z| and phase angle in the Bode plot were the largest, and the corrosion of 2205 DSS was the smallest. With the increase of the solution temperature, the impedance modulus |Z|, the radius of the capacitive arc, and the solution resistance decreased, and in addition, the phase angle also reduced from 79 to 58 Ω at the mid-frequency region and presented a wider peak, a dense inner layer and a sparse (porous) outer layer was mainly characteristics of the inhomogeneous passivation film^28^. Therefore, as the temperature increased, the dissolution and rupture of the passivation film formed on the surface of the metal substrate weakened the protective properties of the substrate, and the corrosion resistance of the material became worse^29^.

**Figure 3:**
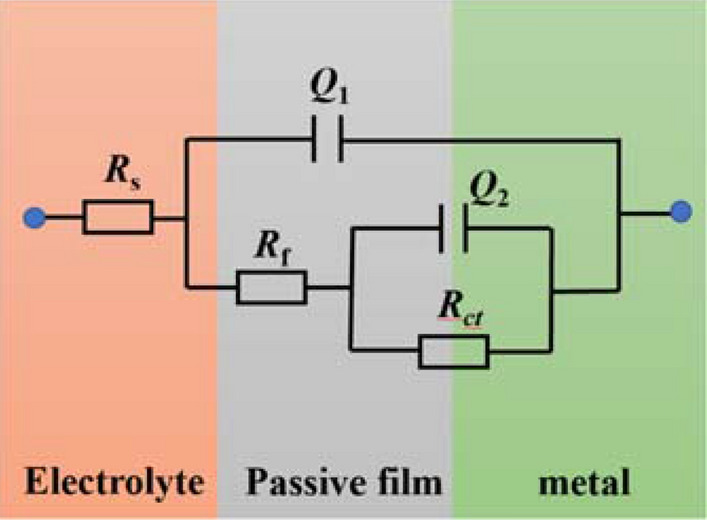
Equivalent circuit diagram of EIS curve fitting.

The impedance spectrum data were fitted using ZSimDeme software, and the fitted equivalent circuit was shown in Fig. 3^30^ where *R*_s_ is the simulated solution resistance, *Q*_1_ is the film layer capacitance, *R*_f_ is the generated passivation film resistance, *Q*_2_ is the bilayer capacitance, and *R*_ct_ represents the charge transfer resistance. As shown of the fitting results in Table 3, the value of n_1_ decreased from 0.841 to 0.769 as the temperature of the simulated solution increased, indicating that the gap between the bilayer capacitance increased and the denseness decreased. The charge transfer resistance *R*_ct_ gradually decreased from 2.958 × 10^14^ to 2.541 × 10^3^ Ω·cm^2^, indicating that the corrosion resistance of the material gradually decreased. The solution resistance *R*_s_ reduced from 2.953 to 2.469 Ω·cm^2^, while the passivation film capacitive resistance Q_2_ decreased from 5.430 × 10^–4^ to 1.147 × 10^–3^ Ω·cm^2^, the solution conductivity increased, the stability of the passivation film decreased, and the activity of aggressive ions (Cl^−^, SO_4_^2−^, etc.) in the solution increased, accelerating the damage to the passivation film^31^. This caused a decrease in the film resistance *R*_f_ formed on the surface of duplex stainless steel (from 4662 to 849 Ω·cm^2^) and a decrease in the polarization resistance *R*_p_ (*R*_ct_ + *R*_f_).

**Table 3:** EIS fitting results of 2205 DSS in simulated solution containing 100 g/L Cl^-^ and saturated CO_2_ at different temperatures.

Temperature/(°C)	*R*_s_/(Ω·cm^2^)	Q_1_/(F·cm^−2^)	n_1_	*R*_f_/(Ω·cm^2^)	Q_2_/(F·cm^−2^)	n_2_	*R*_ct_/(Ω·cm^2^)
30	2.953	5.338 × 10^–4^	0.841	4662	5.430 × 10^–4^	0.361	2.958 × 10^14^
45	2.733	5.867 × 10^–5^	0.827	4025	1.378 × 10^–3^	0.382	7.761 × 10^10^
60	2.522	8.904 × 10^–5^	0.792	1429	8.192 × 10^–4^	0.448	1.207 × 10^4^
75	2.469	1.101 × 10^–4^	0.769	849	1.147 × 10^–3^	0.466	2.541 × 10^3^

Therefore, the solution temperature affected the corrosion resistance of 2205 DSS, when the solution temperature was lower, the cathode and anode reaction process occured in the presence of Fe^2+^, prompting the anode rapid dissolution of corrosion, the passivation film formed on its surface was more complete and higher density, the charge transfer resistance between the solution was larger, so that the metal substrate dissolution became slower, showing better corrosion resistance. As the temperature of the solution increased, the charge transfer resistance *R*_ct_ decreased, the reaction rate between the ions in solution was accelerated, and the diffusion rate of the aggressive ions increased, making the original generated corrosion products from the surface of the metal substrate, thus a more sparse passivation film on the surface of the substrate was re-formed and the protective properties to the substrate was weakened^32^.

### Dynamic potential polarization curve

**Figure 4:**
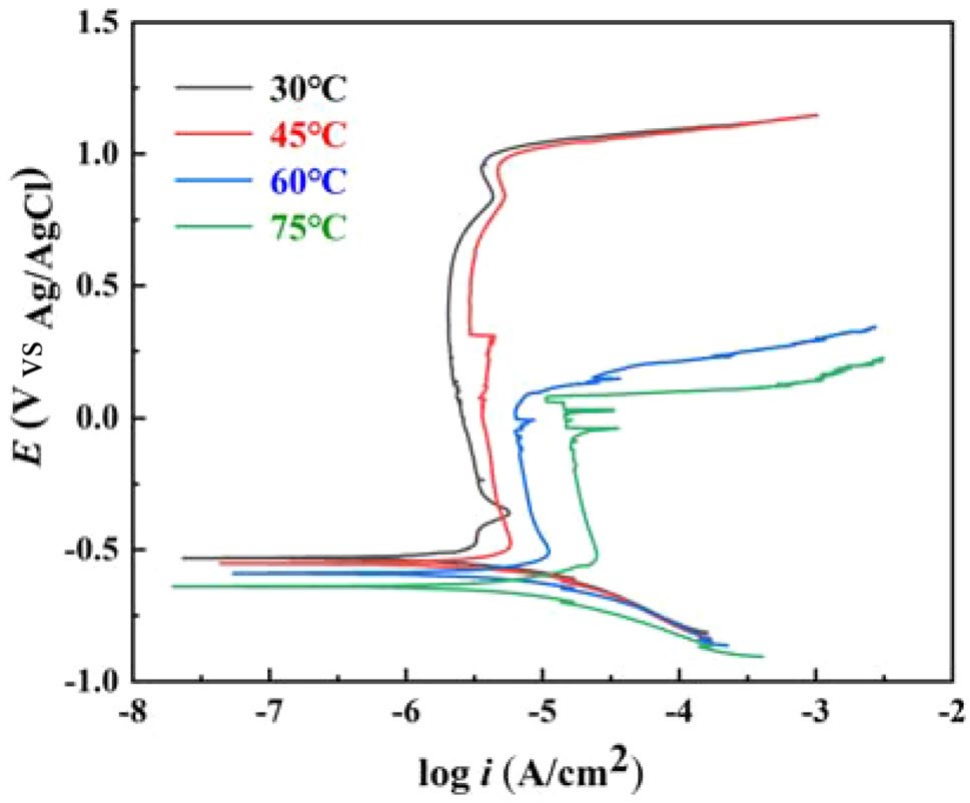
Dynamic potential polarization curve of 2205 DSS at different temperatures.

Figure 4 shows the dynamic potential polarization curves of 2205 DSS in simulated solution containing 100 g/L Cl^−^ and saturated CO_2_ at different temperatures. As can be seen from the figure, when the potential was between − 0.4 and 0.9 V, the anodic curves at different temperatures had obvious passivation zones, while the self-corrosion potential was about − 0.7 to − 0.5 V. The potential corresponding to the anodic curve was usually called the pitting potential (*E*_b_ or *E*_tra_) when the current density increased to 100 μA/cm^2^^33^. With the increasing temperature, the passivation interval decreased, the self-corrosion potential decreased, the corrosion current density tended to increase, and the polarization curve moved down to the right, indicating that the activity of the film layer of 2205 DSS formed in simulated solution containing 100 g/L Cl^−^ and saturated CO_2_ enhanced, pitting corrosion sensitivity increased, and it is easily destroyed by aggressive ions, resulting in an increase in corrosion of the metal matrix and decrease in corrosion resistance.

As can be seen from Table 4, the corresponding overpassivation potential decreased slightly when the temperature increased from 30 to 45 °C, but the corresponding dimensional passivation current density increased significantly, indicating that the protective effect of the passivated film increased with temperature under these conditions. When the temperature reached 60 °C, the corresponding pitting breakdown potential decreased significantly, and this trend became more obvious with increasing temperature. It is noteworthy that a significant current transient peak appeared in the plot at 75 °C, indicating the present of sub-stable pitting on the surface of the specimen.

**Table 4:** Fitting results of polarization curve of 2205 DSS at different temperatures.

Temperature/(°C)	*E*_corr_/(V)	*E*_tra_ or *E*_b_/(V)	*i*_corr_/(A·cm^−2^)
30	− 0.532	1.094	1.482 × 10^–6^
45	− 0.553	1.085	1.934 × 10^–6^
60	− 0.580	0.212	2.556 × 10^–6^
75	− 0.621	0.098	2.893 × 10^–6^

Therefore, as the solution temperature increased, the amount of dissolved oxygen in the solution decreased, and then the pH value of the film surface decreased and the stability of the passivated film reduced. In addition, the higher the solution temperature, the higher the activity of aggressive ions in the solution, the higher the rate of damage to the substrate surface film layer. The formation of oxides in the film layer easily fell off, which reacted with the cations in the film layer to generate soluble compounds, increasing the chance of pitting corrosion. Because the re-generated film layer was relatively loose, the protection to the substrate was lower, and the corrosion of the metal substrate increased. The results of the dynamic potential polarization test were consistent with the results of impedance spectroscopy.

### Potentiostatic polarization curve

**Figure 5:**
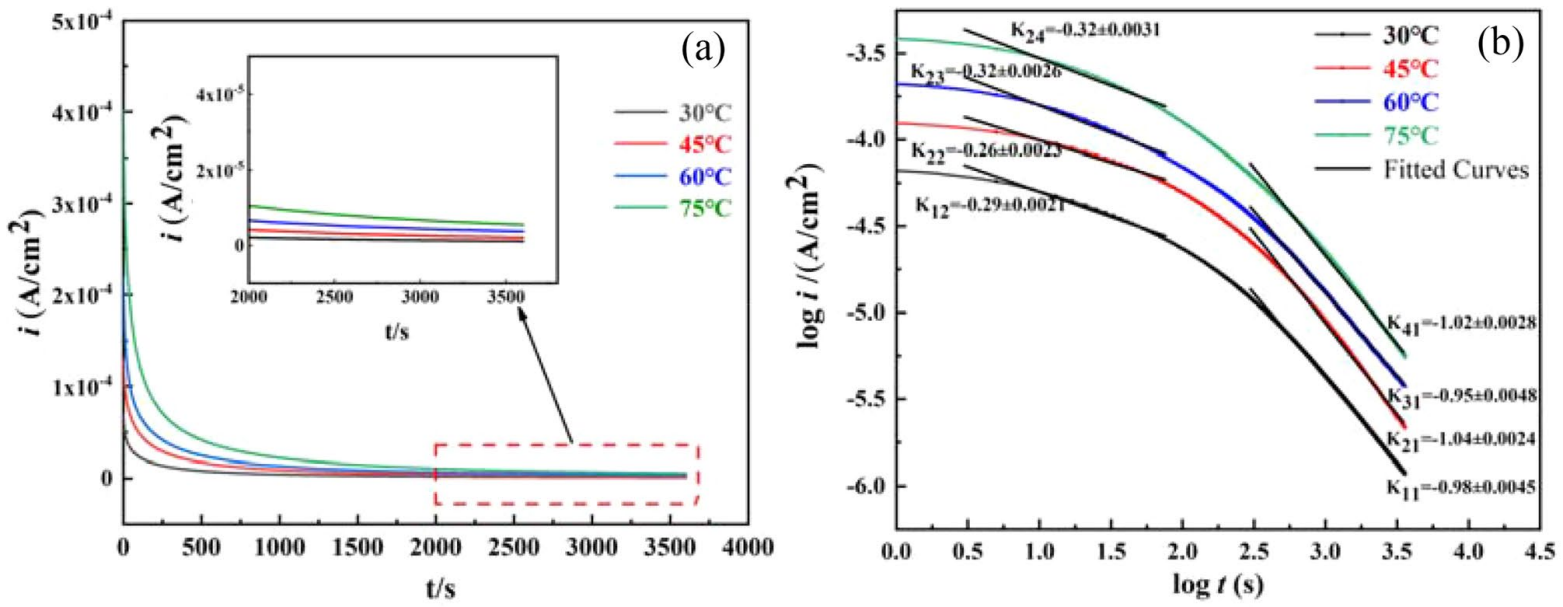
Constant potential polarization curves of 2205 DSS at the same film formation potential vs different temperatures. (**a**) Current density versus time, (**b**) log*i*- log*t* for passivation film growth.

Figure 5a shows the *I*-*t* curve of 2205 DSS in simulated solution containing 100 g/L Cl^−^ and saturated CO_2_. The change of passivation current density with time was obtained after polarization at different temperatures for 1 h at − 300 mV (vs.Ag/AgCl) potential. It can be seen that the trend of the passivation current density of 2205 DSS at different temperatures in the same potential was basically same, and the trend gradually decreased and became smooth with the increase of time. While with the gradually increase of temperature, the passivation current density of 2205 DSS increased, which is consistent with the polarization results, further indicating that the protective property of the film layer to the metal substrate decreased with the increase of the solution temperature.

To investigate the relationship between passivation current density and time for the same film-forming potential at different temperatures as shown in (1)^34^:

1 $${{i = 10}}^{{ - {{(A + K \text{log}t)}}}}$$

where *i* is the passivation current density at the film-forming potential, A/cm^2^. *A* is the working electrode area, cm^2^. K is the slope of the fit to the *i*-*t* curve. *t* is the time, s. 

Figure 5b shows the log*I* and log*t* curves of 2205 DSS at different temperatures in the same film-forming potential. According to the literature^35^, when the slope of the line was K =  − 1, the film layer formed on the surface of the substrate was denser and had better corrosion resistance to the metal substrate. While when the slope of the line was K =  − 0.5, the film layer generated on the surface was loose, and contained many small holes, which had poor corrosion resistance to the metal substrate. It can be seen that at 30 °C, 45 °C, 60 °C and 75 °C, the structure of the film layer was transformed from dense to loose porosity according to the fitted linear slope. According to the point defect model (PDM)^36,37^, it is known that the potential applied during the test did not affect the current density, indicating that the temperature directly affected the measurement of the anode current density during the test, thus the current density increased and the corrosion resistance of 2205 DSS decreased with the increase of the solution temperature.

### Mott-Schotty curves

The nature of the semiconductor of the film layer formed on DSS influenced its corrosion resistance^38^, and the type of semiconductor and the carrier density of the film layer affected the rupture of the film layer and pitting of DSS^39,40^, where the capacitance C and potential E of the film layer met the M-S relationship, the semiconductor space charge was calculated^41^ as follows:

Space charge of p-type semiconductor:

2 $$C^{ - 2} = - \frac{2}{{\varepsilon \varepsilon_{0} eN_{A} }}\left( {E - E_{fb} - \frac{KT}{e}} \right)$$

Space charge of n-type semiconductor:

3 $$C^{ - 2} = - \frac{2}{{\varepsilon \varepsilon_{0} eN_{D} }}\left( {E - E_{fb} - \frac{KT}{e}} \right)$$

where *ε* is the dielectric constant of passivation film at room temperature, taking 12^30^; *ε*_0_ is the vacuum permittivity, taken as 8.85 × 10^−14^F/cm; *E* is the sub charge (1.602 × 10^−19^C) ; *N*_D_ is the donor density of n-type semiconductor, cm^−3^; *N*_A_ is the acceptor density of p-type semiconductor, cm^−3^; EFB is the flat band potential, V; *K* is the boitzmann constant, taken as 1.38 × 10^–23^ J/K; *T* is the temperature, K.

The slope and intercept of the fitted line can be calculated by fitting a linear partition to the measured M-S curve, and the applied concentration (*N*_D_), the received concentration (*N*_A_) and the flat-band potential (*E*_fb_)^42^.

**Figure 6:**
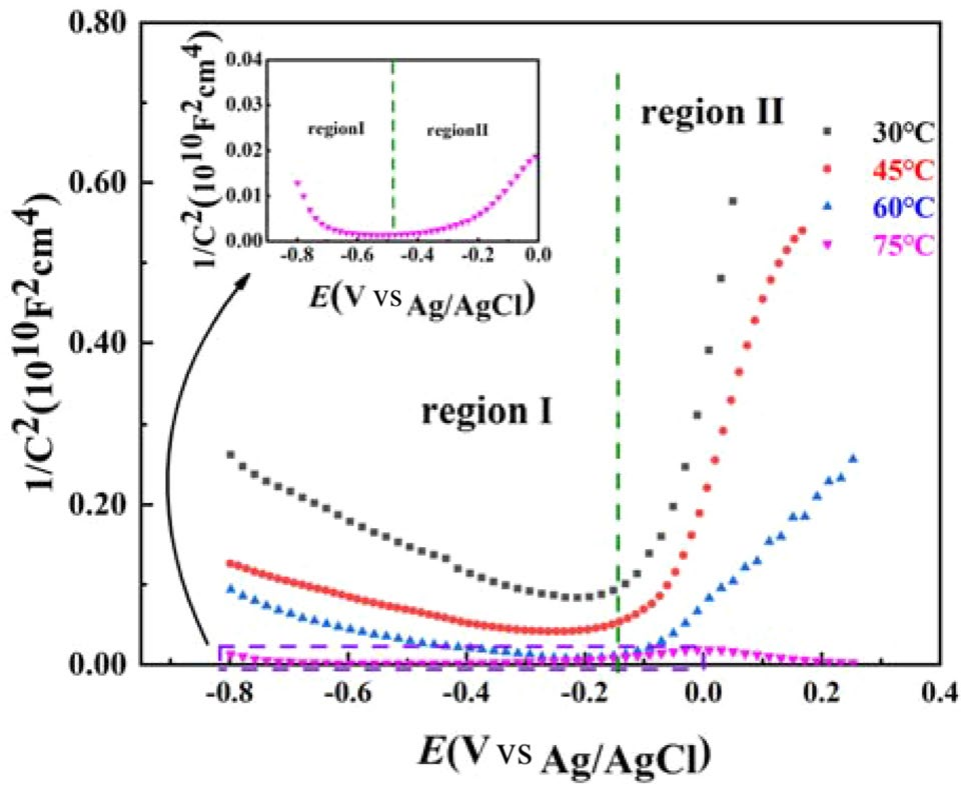
Mott-Schottky curve of 2205 DSS after polarization for 1 h.

Figure 6 shows the Mott-Schottky curve of the surface film layer of 2205 DSS in simulated solution containing 100 g/L Cl^−^ and saturated CO_2_ after film formation at a potential of (− 300 mV) for 1 h. It can be seen that the film layers formed at different temperatures presented the characteristics of n + p type bipolar semiconductor. n-type semiconductors have selective characteristics for anions in solution, which can prevent cations in stainless steel from diffusing through the passivation film into the solution, while p-type semiconductors have selective characteristics for cations, which can prevent aggressive anions in solution from crossing the passivation film into the substrate surface^26^. It can also be seen that there was a gentle transition zone between the two fitted curves, in which the film was in a flat-band state, and the flat-band potential *E*_fb_ can be used to determine the energy band position of the semiconductor and judge its electrochemical stability^43^.

The sender concentration (*N*_D_) and the receiving density (*N*_A_) as well as the flat-band potential *E*_fb_ were calculated from the results of the M-S curve fitting as shown in Table 5, and the overall sender and receiving concentrations were in the range of 10^20^–10^23^ cm^−3^, which is in the same order of magnitude as the calculated results of other studies^44^. The applied host current density mainly characterized the point defects in the space charge layer, the pitting potential of the passivated film, and the larger the applied host concentration, the easier the film layer is broke, and the easier the matrix is corroded^45^. Moreover, with the gradually increase of the solution temperature, the sender concentration *N*_D_ in the film layer increased from 5.273 × 10^20^ cm^−3^ to 1.772 × 10^22^ cm^−3^, the host concentration *N*_A_ increased from 4.972 × 10^21^ to 4.592 × 10^23^ cm^−3^, the flat-band potential increased from 0.021 to 0.753 V, the number of carriers in the solution increased, the interionic reaction process in the solution intensifies, and the stability of the film layer decreased. The smaller the absolute value of the slope of the fitted straight line with increasing solution temperature, the greater the carrier density in the solution, the higher the diffusion rate between ions, the more ionic vacancies on the surface of the film layer, and thus the lower the stability and corrosion resistance of the metal substrate^46,47^.

**Table 5:** Fitting results of Mott Schottky curve (donor concentration *N*_D_ and acceptor concentration *N*_A_).

Temperature/°C	*N*_D1_/cm^−3^	*N*_D2_/cm^−3^	*N*_A1_/cm^−3^	*N*_A2_/cm^−3^	*E*_fb_/Vs._Ag/AgCl_
30	3.742 × 10^21^	5.273 × 10^20^	4.972 × 10^21^	7.634 × 10^21^	0.021
45	7.581 × 10^21^	8.974 × 10^20^	2.373 × 10^22^	7.791 × 10^22^	0.064
60	9.743 × 10^21^	2.145 × 10^21^	5.293 × 10^22^	9.013 × 10^22^	0.235
75	1.262 × 10^23^	1.772 × 10^22^	4.592 × 10^23^	3.914 × 10^23^	0.753

### Component analysis

**Figure 7:**
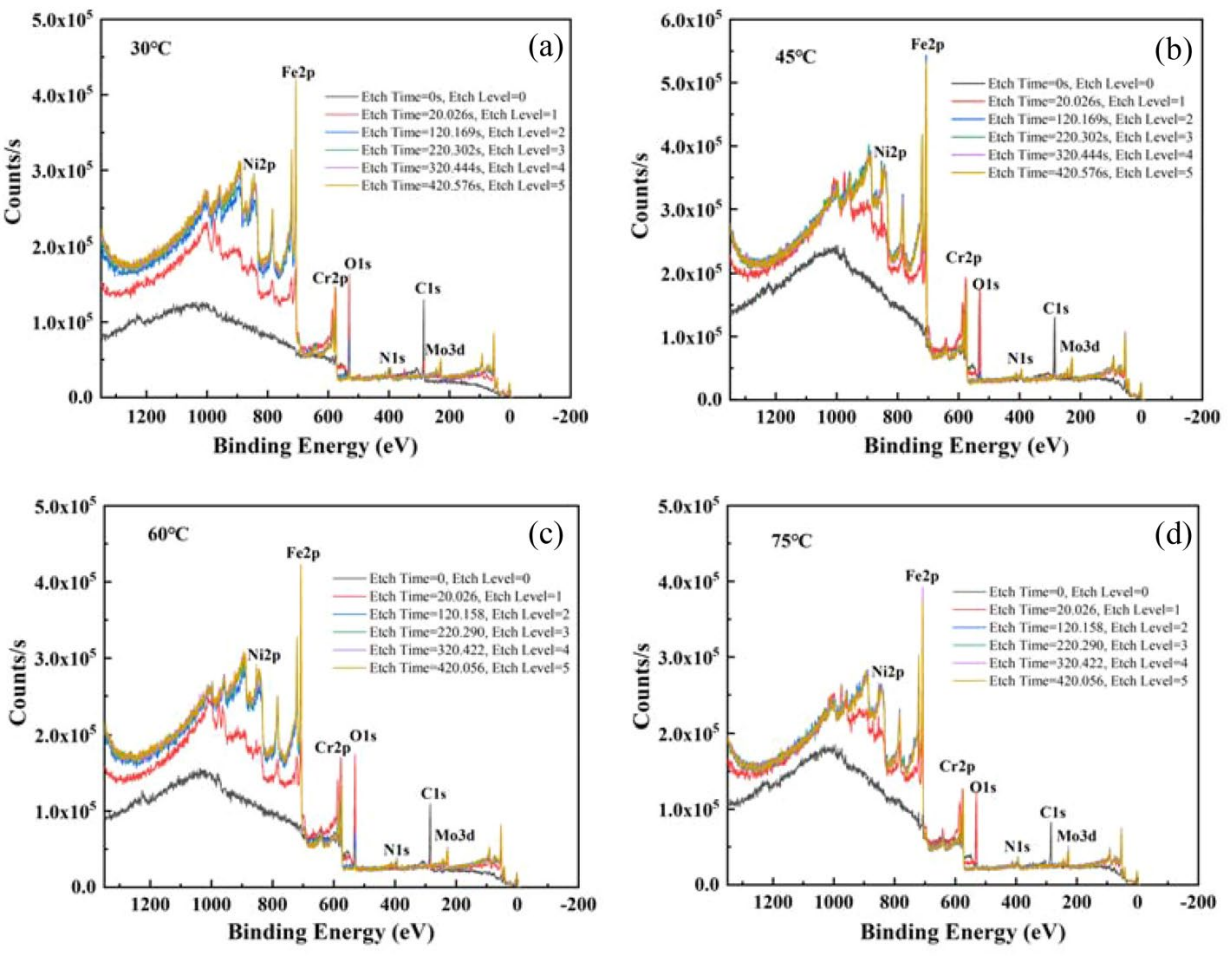
XPS full spectrum of 2205 DSS formed at different temperatures. (**a**) 30 °C, (**b**) 45 °C, (**c**) 60 °C, (**d**) 75 °C.

The chemical composition of the film layer has a significant impact on the stability of metal cations and semiconductor properties, and the change of temperature has an important effect on the film layer generation of stainless steel. Figure 7 shows the full XPS scan spectrum of the surface film layer of 2205 DSS in simulated solution containing 100 g/L Cl^−^ and saturated CO_2_. The main elements in the film layer formed by the chips at different temperatures were basically same, and the main components of its film layer were Fe, Cr, Ni, Mo, O, N and C. Therefore, the main components of the film layer ware Cr oxides, Fe oxides and hydroxides, along with a small amount of Ni and Mo oxides.

**Figure 8:**
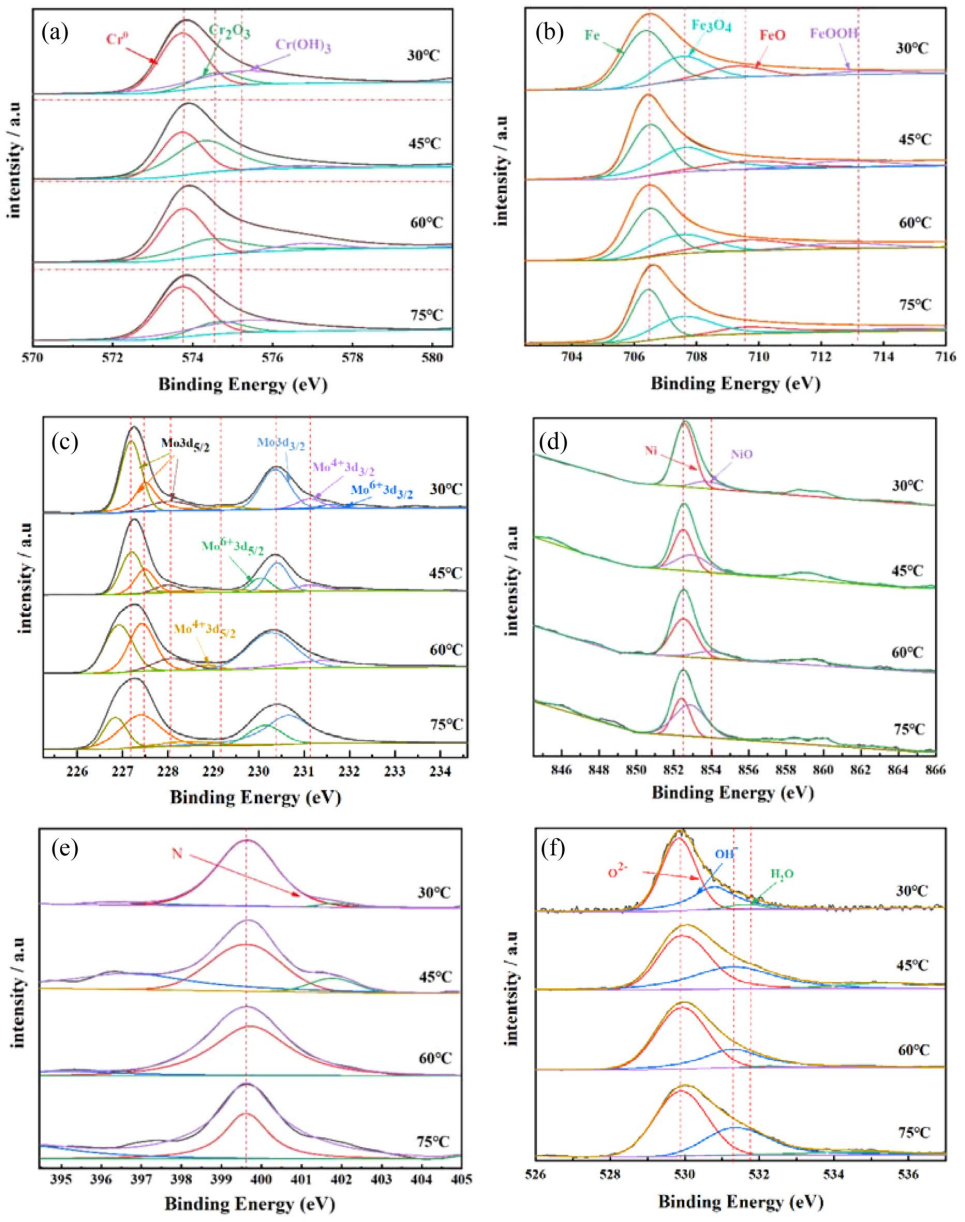
2205 DSS at different temperatures; (**a**) Cr2p, (**b**) Fe2p, (**c**) Mo3d, (**d**) Ni2p, (**e**) N1s, (**f**) O1s.

The main components of the film were related to the thermodynamic properties of the compounds in the passivated film. Based on binding energy of main elements in the film layers as shown in Table 6, it can be seen that the characteristic spectral peak positions of Cr2p3/2 were divided into three peaks consisting of metallic Cr^0^ (573.7 ± 0.2 eV), Cr_2_O_3_ (574.5 ± 0.3 eV) and Cr(OH)_3_ (575.4 ± 0.1 eV), as shown in Fig. 8a, where the oxides formed by the element Cr were the main component in the film and had an important role in the corrosion resistance of the film as well as in its electrochemical characteristics. The relative peak intensity of Cr_2_O_3_ was higher than that of Cr(OH)_3_ in the film layer. However, with the increase of solution temperature, the relative peaks of Cr_2_O_3_ gradually weakened, while the relative peaks of Cr(OH)_3_ gradually increased, indicating that the main Cr^3+^ in the film layer obviously changed from Cr_2_O_3_ to Cr(OH)_3_ with the increase of solution temperature.

**Table 6:** Binding energy of main elements in the film layers.

Element	Peak	Species/binding energy(eV)
Cr	2p3/2	Cr(met)/573.7 ± 0.2; Cr_2_O_3_/574.5 ± 0.3; Cr(OH)_3_/575.4 ± 0.1
Fe	2p3/2	Fe(met)/706.4 ± 0.2; Fe_3_O_4_/707.5 ± 0.2; FeO/709.5 ± 0.1 FeOOH/713.1 ± 0.3
Mo	3d5/2	Mo(met)/227.5 ± 0.3; Mo^4+^/228.9 ± 0.2; Mo^6+^/229.4 ± 0.3
	3d3/2	Mo(met)/230.4 ± 0.1; Mo^4+^/231.5 ± 0.2; Mo^6+^/232.8 ± 0.1
Ni	2p3/2	Ni(met)/852.4 ± 0.2; NiO/854.1 ± 0.2
N	1 s	N/399.6 ± 0.3
O	1 s	O^2−^/529.7 ± 0.2, OH^−^/531.2 ± 0.2; H_2_O/531.8 ± 0.3

The binding energy of the characteristic spectral peak positions of Fe2p3/2 was mainly composed of four peaks of the metallic state Fe^0^ (706.4 ± 0.2 eV), Fe_3_O_4_ (707.5 ± 0.2 eV), FeO (709.5 ± 0.1 eV) and FeOOH (713.1 ± 0.3 eV), as shown in Fig. 8b, and Fe mainly present in the generated film layer as Fe^2+^ and Fe^3+^. In the peaks of lower binding energy, Fe^2+^ of FeO was predominant of Fe(II); while in the peaks of higher binding energy, While Fe(III) compounds of Fe_3_O_4_ and FeOOH were predominant^48,49^. The relative peak intensity of Fe^3+^ was higher than that of Fe^2+^, but the relative peak intensity of Fe^3+^ decreased with increasing solution temperature, while the relative peak intensity of Fe^2+^ enhanced, indicating that the main substance in the film layer changed from Fe^3+^ to Fe^2+^ with increasing solution temperature.

The characteristic spectral peaks of Mo3d5/2 mainly consisted of two peak positions, Mo3d5/2 and Mo3d3/2^43,50^, where Mo3d5/2 included metallic Mo (227.5 ± 0.3 eV), Mo^4+^ (228.9 ± 0.2 eV), and Mo^6+^ (229.4 ± 0.3 eV); while Mo3d3/2 also contained metallic Mo (230.4 ± 0.1 eV), Mo^4+^ (231.5 ± 0.2 eV) and Mo^6+^ (232.8 ± 0.1 eV), as shown in Fig. 8c, so Mo element presented in the film layer in the above three valence states. The binding energies of the characteristic spectral peaks of Ni2p3/2 were composed of Ni^0^ (852.4 ± 0.2 eV), and NiO (854.1 ± 0.2 eV), as shown in Fig. 8d, respectively. The characteristic peaks of N1s consisted of N (399.6 ± 0.3 eV), as shown in Fig. 8e. The characteristic peaks of O1s included O^2−^ (529.7 ± 0.2 eV), OH^−^ (531.2 ± 0.2 eV) and H_2_O (531.8 ± 0.3 eV), as shown in Fig. 8f, which mainly played the role of connecting bonds in the film layer, and the main components of the surface film layer were (OH^−^ and O^2−^), which were mainly used in the film layer for the oxidation or hydroxide of Cr and Fe. The relative peak intensity of OH^-^ increased significantly when the temperature increased from 30 to 75 °C. Therefore, the main material composition of O^2−^ in the film layer changed from O^2−^ to OH^−^ and O^2−^ with the increase of temperature.

### Analysis of corrosion morphology

**Figure 9:**
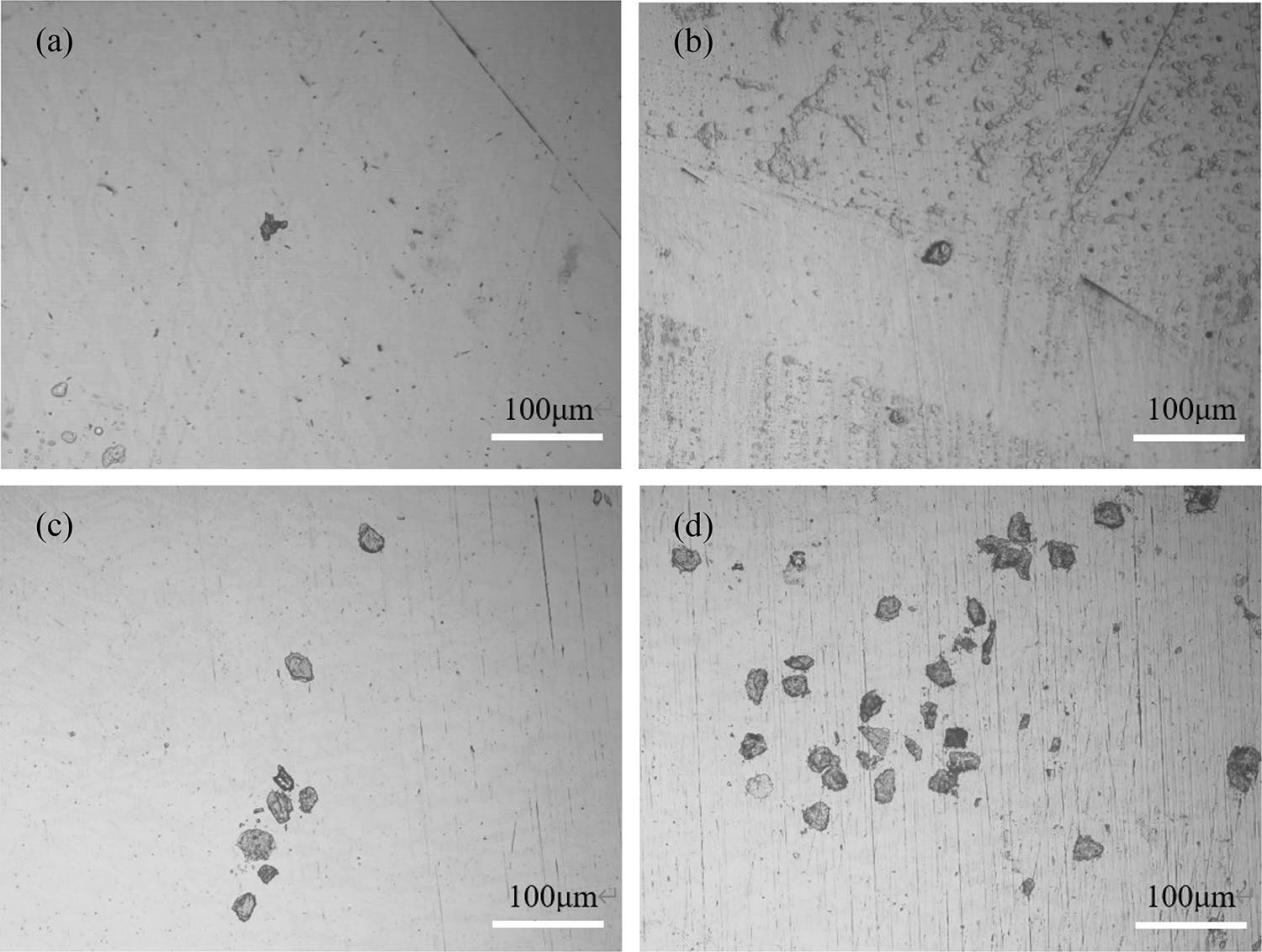
Corrosion profile of 2205 DSS in simulated solution containing 100 g/L Cl^−^ and saturated CO_2_ at different temperatures (**a**) 30 °C, (**b**) 45 °C, (**c**) 60 °C, (**d**) 75 °C.

Figure 9 shows the microscopic morphologies of the surface of 2205 DSS specimen after dynamic potential polarization in simulated solution containing 100 g/L Cl^−^ and saturated CO_2_. It can be seen that the surface of the specimen after polarization at different temperatures had different degrees of corrosion pits, the surface of the specimen at the part without corrosion pits was relatively flat, and there were no obvious corrosion traces, indicating that pitting corrosion of 2205 DSS at different temperatures in the solution containing aggressive ions occured, and with the increase in solution temperature, the surface of the substrate appears more serious corrosion. The number of pitting pits per unit area and the depth of corrosion pits increased.

Therefore, the increase in temperature would increase the activity of the components of DSS, the activity of aggressive ions in the corrosive medium increased, causing a certain degree of damage to the surface of the specimen, and prompting an increase in pitting activity and the formation of pitting pits, the corrosion product generation rate became faster, thus the corrosion resistance of the material reduced^51–55^.

**Figure 10:**
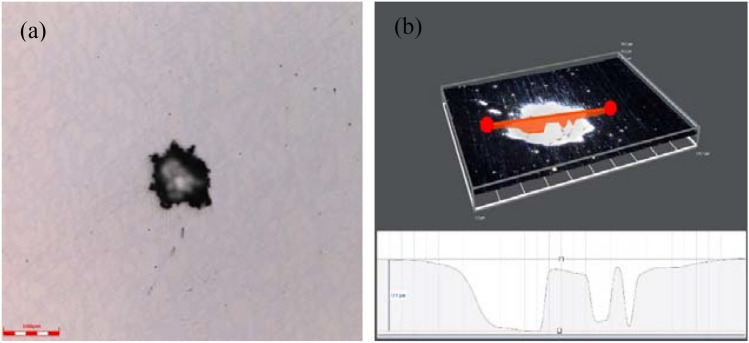
Pitting morphology of 2205 DSS (**a**) Pitting micro morphology, (**b**) Pitting depth.

Figure 10 shows the pitting morphologies and pitting depth of 2205 DSS specimen after polarization using a super depth of field optical digital microscope. From Fig. 10a, it can be seen that there were also smaller corrosion pits around the large pitting pits, indicating that the passivation film on the surface of the specimen was partially destroyed under this current density to form pits, and the maximum pitting depth was 12.9 μm, as shown in Fig. 10b.

## Corrosion mechanism

DSS exhibited better corrosion resistance, the main reason was that the film formed on the steel surface had well protection in solution, Mott-schottky and according to XPS results mentioned above and related literatures^13,56–58^, the film reacted anodically mainly through the following oxidation processes of Fe and Cr.

4 $${\text{Fe}} + {\text{2OH}}^{ - } \to {\text{Fe(OH)}}_{{2}} + {\text{ 2e}}^{ - }$$

5 $${\text{2Fe(OH)}}_{{2}} + {\text{2OH}}^{ - } \to {\text{Fe}}_{{2}} {\text{O}}_{{3}} + {\text{3H}}_{{2}} {\text{O}} + {\text{2e}}^{ - }$$

6 $${\text{Cr}} + {\text{3OH}}^{ - } \to {\text{Cr(OH)}}_{{3}} + {\text{3e}}^{ - }$$

7 $${\text{Cr(OH)}}_{{3}} + {\text{Cr}} + {\text{3OH}}^{ - } \to {\text{Cr}}_{{2}} {\text{O}}_{{3}} + {\text{3H}}_{{2}} {\text{O + 3e}}^{ - }$$

Fe^2+^ is easy to dissolve and deposit at the interface between the film and solution^53^, and the cathodic reaction process is as follows:

8 $${\text{O}}_{{2}} + {\text{2H}}_{{2}} {\text{O}} + {\text{4e}}^{ - } \to {\text{4OH}}^{ - }$$

In the corroded state, a bilayer structured film layer was formed, which mainly consisted of the inner layer of Fe and Cr oxides and the outer layer of hydroxides^59^, with ions usually growing in the empty spaces of the film layer. The chemical composition of the passivation film is related to its semiconductor properties, as shown by the mott-schottky curve, which shows that the composition of the passivation film is of the n + p type with bipolar characteristics. The XPS results show that the outer layer of the passivation film is mainly composed of Fe oxides and hydroxides showing n-type semiconductor characteristics, while the inner layer is mainly composed of Cr oxides and hydroxides showing p-type semiconductor characteristics.

2205 DSS presented high resistance properties due to its high Cr content^17,54^, and also presented different degrees of pitting corrosion due to its microscopic galvanic corrosion between duplex tissues^55^. Pitting was one of the most common types of corrosion in DSS, and temperature was one of the important factors affecting the pitting behavior, and also had an effect on both thermodynamic and kinetic reaction processes of DSS^60,61^. Generally, in the simulated solution with high concentration Cl^−^ and saturated CO_2_, the temperature also had an influence on the formation of pitting and crack initiation in stress corrosion cracking of DSS, and the critical pitting temperature was determined to evaluate the corrosion resistance of the material, which reflected the sensitivity of the metal matrix to temperature and was usually used as an important reference index for material selection in engineering applications. 2205 DSS had an average critical pitting temperature of 66.9 °C in the simulated solution, which was 25.6 °C higher than that of super 13Cr stainless steel in 3.5 wt% NaCl, although the maximum depth of pit had reached 12.9 μm^62^. The electrochemical results further confirmed that the horizontal region of phase angle and frequency narrowed with increasing temperature, with the phase angle value decreasing from 79° to 58°, the impedance mode value |Z| decreasing from 1.26 × 10^4^ to 1.58 × 10^3^ Ω·cm^2^; the charge transfer resistance *R*_ct_ decreased from 2.958 × 10^14^ to 2.541 × 10^3^ Ω·cm^2^, the solution resistance Rs reduced from 2.953 to 2.469 Ω·cm^2^, film layer resistance *R*_f_ from 5.430 × 10^−4^cm^2^ to 1.147 × 10^−3^cm^2^. Corrosion solution conductivity enhanced, the stability of the metal substrate film layer is reduced, easy to dissolve rupture. The self-corrosion current density increased from 1.482 to 2.893 × 10^–6^ A·cm^−2^, and the self-corrosion potential decreased from − 0.532 to − 0.621 V. It can be seen that the change of temperature has some influence on the integrity and denseness of the film layer.

In contrast, the high concentration Cl^−^ and saturated CO^2^ solution, as the temperature increased, the adsorption capacity of Cl^−^ on the surface of the passivation film gradually increase, the stability of the passivation film becomes less stable, the protection of the substrate becomes weaker, and the sensitivity to pitting corrosion increases. At the same time, the activity of corrosive ions in solution increased and the oxygen content decreased, the surface film layer of the eroded material was difficult to repair quickly, which provided more favorable conditions for further adsorption of aggressive ions on the surface the pitting potential of the material decreases^63^. Robinson et al^64^ showed that when the temperature of the solution increased, the growth rate of pitting accelerated, the rate of ion diffusion within the solution increased, and when the temperature raise to 65 °C, the dissolution of oxygen in solutions containing Cl^−^ ions decreased, the cathodic reaction process slowed down, and the rate of pitting formation decreased. Han^20^ explored the effect of temperature on the corrosion behavior of 2205 duplex stainless steel in CO_2_ environment, and the results showed that the increase in temperature increases the number of corrosion products and the area of craters on the surface of the material. Also when the temperature was increased to 150 °C, the surface oxide film broke and the crater density was the highest. Liu^4^ explored the effect of temperature on the corrosion behavior of 2205 duplex stainless steel from passivation to activation in a geothermal environment containing CO_2_. Their results show that when the test temperature is less than 150 °C, the generated film layer exhibits amorphous structural properties and contains a Ni-rich layer at the internal interface, while when the temperature is 300 °C, the generated corrosion products are nanopolycrystalline FeCr_2_O_4_, CrOOH and NiFe_2_O_4_.

**Figure 11:**
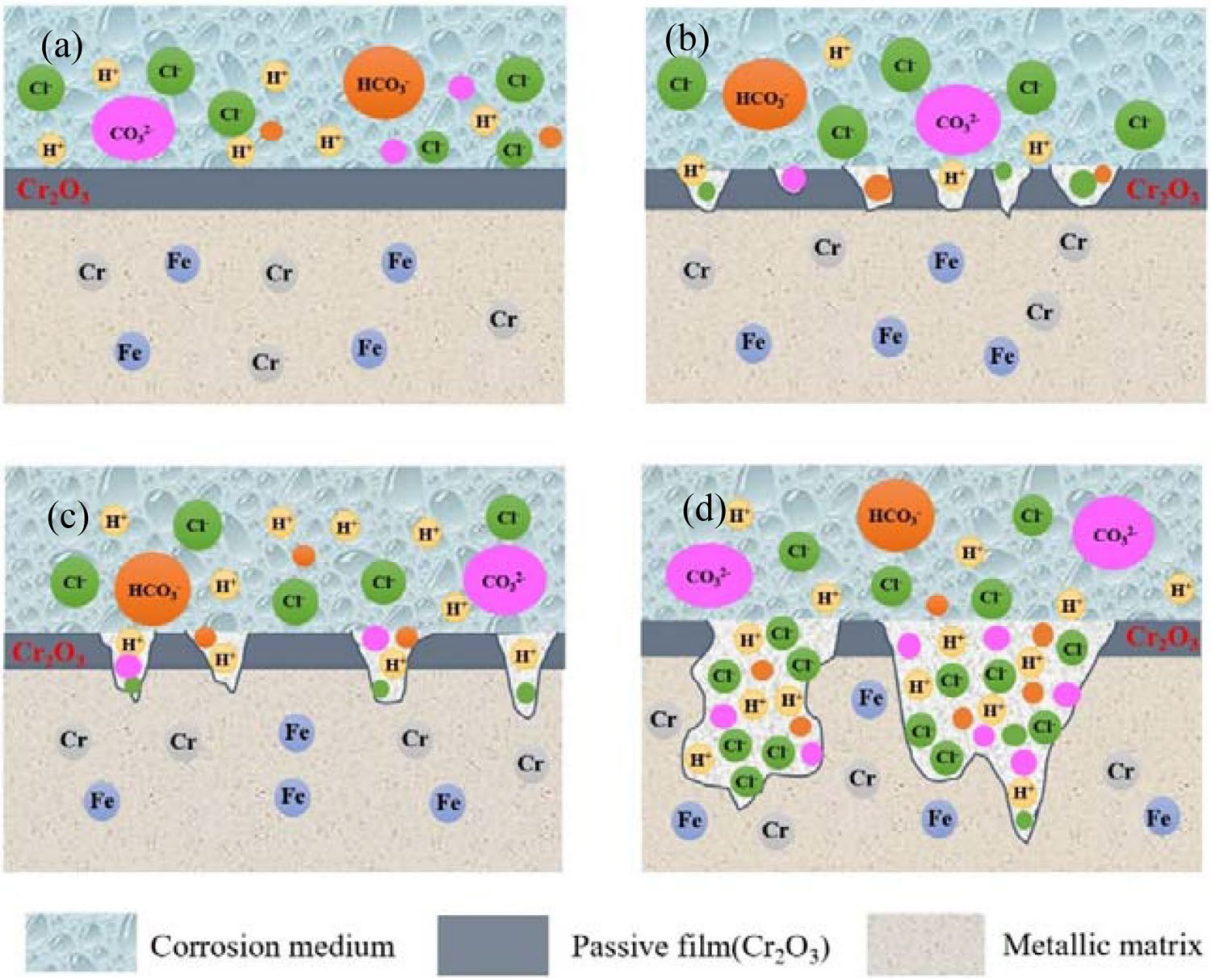
Corrosion process of 2205 DSS and formation and fracture of new film. (**a**) Process 1, (**b**) Process 2, (**c**) Process 3, (**d**) Process 4.

Figure 11 shows the corrosion processes and the schematic diagram of film formation and rupture of 2205 DSS. 2205 DSS has formed a passivated film in the atmosphere before service, and once immersed in the simulated solution medium with high Cl^−^ and CO_2_ containing solutions, its surface is rapidly surrounded by a variety of aggressive ions (Cl^−^, CO_3_^2−^, etc.). J. Banas^65^ concluded that in the environment where there is also CO_2_, the stability of the passivation film on the material surface decreases with time and the carbonic acid generated tends to increase the conductivity of the ions in the passivation film and accelerate the dissolution of the passivation film. Thus the film layer on the surface of the specimen was in a dynamic equilibrium stage of dissolution and repassivation^66^, Cl^−^ reduces the generation rate of the surface film layer appeared as tiny pitting pits in the adjacent areas of the film surface as shown in Fig. 11a and b, while tiny and unstable corrosion pits appeared, and the activity of the corrosive ions in the solution on the film layer enhanced and the depth of tiny unstable pits deepened with the increase in temperature, until the film layer is completely penetrated, as shown in Fig. 11c. When the solution medium temperature increased further, the content of dissolved CO_2_ in the solution is accelerated, resulting in a lower pH in the solution, the density of tiny unstable corrosion pits on the surface of DSS increased, the depth of the original corrosion pits expanded and deepened, the thickness of the passivation film on the surface of the specimen was thin, and the passivation film was more prone to pitting corrosion as shown in Fig. 11d. And the electrochemical results further confirmed that the change of temperature has some influence on the integrity and denseness of the film layer. Therefore, it can be seen that the corrosion in saturated CO_2_ solutions containing high concentration Cl^−^ is significantly different than the corrosiveness in solutions containing low Cl^−^^67,68^.

## Conclusions

1. The average critical pitting temperature of 2205 DSS in simulated solution containing 100 g/L Cl^−^ and saturated CO_2_ was 66.9 °C, the maximum corrosion pit depth was 12.9 μm, and the corrosion resistance of 2205 DSS was weakened and the pitting sensitivity was enhanced with the increase of temperature.
2. 2205 DSS passivation film formed at different temperatures showed n + p type semiconductor properties, with the increase in temperature, the corresponding donor and acceptor carrier density increased, the stability of the passivation film reduced, and then the protective properties of the passivation film was weakened.
3. The main components of the passivation film are Cr oxide and Fe oxide, and the number of corrosion pits on the surface of the specimen increased, the surface pitting activity point was more, and the corrosion pit aperture increased with the increase of the temperature.

## Date availability

The date presented in this study are available on request from the corresponding author.
